# Comparison of sexual distress and sexual avoidance in women with and without genital warts: a case-control study

**DOI:** 10.1186/s12905-026-04263-y

**Published:** 2026-01-28

**Authors:** Atefeh Eslamloueian, Parvin Ghaemmaghami, Mehdi Ghahartars, Roksana Janghorban

**Affiliations:** 1 Student Research Committee, Department of Midwifery, School of Nursing and Midwifery, Shiraz University of Medical Sciences, Shiraz, Iran; 2 School of Nursing and Midwifery, Shiraz University of Medical Sciences, Shiraz, Iran; 3 Department of Dermatology, Molecular Dermatology Research Center, School of Medicine, Shiraz University of Medical Sciences, Shiraz, Iran; 4 Community Based Psychiatric Care Research Center, Department of Midwifery, School of Nursing and Midwifery, Shiraz University of Medical Sciences, Nemazee Square, Zand Blv., Shiraz, Iran

**Keywords:** Genital warts, Human papilloma virus, Sex, Sexual dysfunction, Women

## Abstract

**Background:**

Human papillomavirus is the most common infection transmitted through sexual contact. The occurrence of genital warts (GWs) can affect patients’ sexual life.

**Objective:**

This study was performed to compare sexual distress and sexual avoidance in women with and without GWs.

**Methods:**

This case‒control study was conducted on 136 women with GWs and 136 women without GWs in the dermatology and gynecology clinic of Shahid Faqihi Hospital, Shiraz University of Medical Sciences, Iran, from April to November 2023. Data were collected via a demographic questionnaire, a revised version of the female sexual distress scale, and a sexual avoidance questionnaire and were analysed via Chi square test, independent t-test and a generalized linear model with SPSS software version 22.

**Results:**

The mean scores of sexual distress were 26.16 ± 13.97 and 8.50 ± 6.42 in the case and control groups, respectively. There was a significant difference between the two groups in terms of the sexual distress score (*P* < 0.001). The mean sexual avoidance scores were 9.79 ± 4.00 and 9.48 ± 2.85 in the case and control groups, respectively. There was no statistically significant difference between the two groups in terms of the sexual avoidance score (*P* = 0.465). Women with genital warts (*P* < 0.001), college and above education (0.036), dyspareunia (*P* = 0.003), and higher age at first sexual intercourse (*P* = 0.004) had a significantly higher sexual distress score.

**Conclusions:**

Women with GWs experienced more sexual distress, but their degree of sexual avoidance was not significantly different from that of women without GWs. These results could have important applications in the assessment of women with GWs. Healthcare providers should pay more attention to women’s sexual distress, implement appropriate interventions to diminish it and prevent the occurrence of undesirable psychosexual consequences such as sexual avoidance.

**Supplementary Information:**

The online version contains supplementary material available at 10.1186/s12905-026-04263-y.

## Background

Genital human papillomavirus (HPV) infection is the most common sexually transmitted infection (STI) worldwide and is caused by certain types of viruses [1]. Genital warts (GWs), resulting from abnormal skin cell growth, are caused specifically by HPV types 6 and 11 and are transmitted through skin-to-skin contact, often during sexual intercourse [2]. Most HPV infections, whether low-risk or high-risk, are temporary and typically resolve within 2 years. They are usually asymptomatic and do not have any clinical consequences [3]. Data on the prevalence of GWs among women in different countries vary. In the United States, the Centers for Disease Control and Prevention estimates that an average of 1 in 100 sexually active adults will develop GWs at any given time [4]. In a previous study, the prevalence of this disease among Indian women was reported to be between 1.4% and 25% [5]. In the Philippines, the reported prevalence of GWs among women was 3.39% in 2019 [6]. In Iran, owing to cultural issues and the fact that people seek treatment in private clinics, there are no exact statistics on the prevalence of GWs. However, studies conducted in different centers of the country suggest that the prevalence of GWs is high. For example, a study in Iran revealed that 2.8% of women who visited the clinic with complaints of vaginal infection had GWs [7]. In different societies, the stigma and labels associated with STIs can hinder patient timely diagnosis and treatment, increasing the risk of developing various disorders [8]. GWs, one of the clinical manifestations of the low-risk type of HPV, can negatively impact sexual desire. This is due to the challenges they create in the intimacy of couples, such as weakening their self-confidence and causing doubts about the fidelity of their sexual partner [9–11]. The impact of this virus on women’s sexual life has been reported in the form of sexual dysfunction, psychosexual distress, and a decrease in the quality of sexual life [10–12]. Research conducted on women with GWs both globally and in Iran also indicates the psychological and sexual effects of these lesions [12–15]. Sexual distress is an important and influential indicator of sexual function. It refers to feelings of frustration, worry, and anxiety related to sexual issues. According to the fifth edition of the Diagnostic and Statistical Manual of Mental Disorders, this anxiety must be symptomatic and manifest as disturbances in interpersonal relationships [16]. Research findings in England show that receiving a positive HPV result can cause psychosexual distress, especially in the short term [17]. Sexual avoidance is another type of sexual problem characterized by persistent or recurrent avoidance of any or most sexual interactions or activities that may lead to sexual intimacy with one’s partner [16]. Research indicates that sexual avoidance can significantly impact a person’s life in cases of sexual dysfunction, illness, and health issues, as well as their partner’s sexual difficulties and lack of interest in sex [18]. Additionally, individuals may refuse or avoid sex due to the fear of contracting STIs [19]. Previous studies indicate that a diagnosis of GWs leads to changes in women’s sexual lives, including instability in sexual function, increased anxiety, and general distress [15, 20].

Owing to the increasing prevalence of GWs, the increasing incidence of complications associated with the disease, its impact on sexual health, disruption in couples’ relationships, concerns about body image, the lack of studies in the field of sexual distress in women with GWs and the lack of investigations of the sexual avoidance status of these people, this study aimed to compare sexual distress and sexual avoidance in women with and without GWs.

## Methods

### Study design

This case‒control study was conducted from April to November 2023 at the dermatology and gynaecologic clinic of Shahid Faghihi Hospital at Shiraz University of Medical Sciences, Iran.

### Study population

Participants in both the case and control groups consisted of married Iranian non-pregnant women aged 18 years and above who were attending the gynecology and dermatologic clinics and were sexually active in the past four weeks. The inclusion criteria for both groups included the absence of a current diagnosis of mental disorders and sexual dysfunction according to clinical records and self-report, no use of drugs affecting sexual function, no chronic diseases or cancers, especially cervical cancer, no use of alcohol and drugs, no suffering from other STIs (herpes, syphilis, gonorrhoea, or Human immunodeficiency virus) in women or their spouses, and no spousal occupation with frequent absences from family due to work duties. Additionally, women in case group had GWs that were diagnosed by a dermatologist on the basis of clinical signs in their skin. The exclusion criterion was a lack of willingness to participate in the study after enrollment.

The sample size was calculated using G*Power software (version 3.1.9.7), based on a bivariate correlation (two-tailed) test. Considering an effect size of *r*= − 0.238 derived from a previous similar study [21], a significance level of α = 0.05, and a statistical power of 80% (1–β = 0.80), the minimum required sample size was estimated to be 136 participants.

Sampling was carried out using a convenience purposive method. For the case group, the researcher was present at the dermatology and gynaecologic during the study period and sampled the eligible women who visited for the treatment of genital warts. Diagnosis of genital warts was confirmed by a dermatologist based on the clinical signs of the skin lesions. For the control group, sampling was also conducted among women visiting the clinics who were eligible to participate in the study.

### Data collection

For both groups, sociodemographic information and data about marital status and sexual relationships were collected.

Sexual distress was assessed with the revised version of the female sexual distress scale (FSDS-R), which examines different aspects of sexual distress in women in the past 4 weeks [22]. The scale which was developed by Derogatis et al. consists of 13 items, and all the items are scored on a five-point Likert-type response scale ranging from 0 (never) to 4 (always). The total score can be calculated by adding up the scores of all 13 items ranging from 0 to 52. A higher score indicates greater sexual distress. A score equal to or greater than 11 can effectively differentiate between women with or without sexual distress [22, 23]. In current study, the Cronbach’s alpha coefficient of the scale was found to be 0.817.

Sexual avoidance which was developed by Theiss et al. has a 4-item questionnaire that examines an individual’s avoidance of sexual topics, sexual desires, sexual feelings about each other, and satisfaction with sexual relationships [24]. All the items are measured on a 5-point Likert scale ranging from (1) actively discuss to (5) actively avoid. All the items are reverse-scored, with higher scores indicating greater avoidance [24]. The Cronbach’s alpha coefficient of the questionnaire in the current study was found to be 0.90.

### Ethical issues

The research project was approved by the ethics committee of Shiraz University of Medical Sciences (IR.SUMS.REC.1402.004). Written informed consent was obtained from all the participants. Confidentiality and the right to withdraw from the study were preserved for all participants. To mitigate the risk of social desirability bias, several methodological strategies were implemented. The study ensured complete anonymity and confidentiality through self-administered questionnaires, which participants completed in a private, quiet setting. Completed forms were returned in sealed envelopes to further protect privacy and a unique code was assigned to each questionnaire. Participants were explicitly assured that their responses were anonymous and would remain confidential.

### Data analysis

The mean (standard deviation) was used to describe quantitative data, whereas the frequency (percentage) was used to describe qualitative data. Categorical variables were compared via the chi-square test. Comparisons of the means of variables between two groups were performed via independent t tests. A generalized linear model was performed to control the effect of confounding factors and provide a more accurate comparison between the two groups. The data were analysed via SPSS software, version 22 (SPSS Inc., Chicago, IL, USA). A P value < 0.05 was considered significant.

## Results

The mean time since the onset of GWs was 5.26 ± 6.76 months in the case group. The mean number of intercourse in the month before GWs was 4.38 ± 1.74 and 4.46 ± 1.86 times in the control and the case group, respectively and there is not statistically difference between two groups (*P* value = 0.714). Table 1 compares some demographic characteristics and their sexual relationships between two groups (Table 1).

The results of the chi-square test and independent t test revealed correlations between having GWs and women’s educational status (*P* < 0.001), extramarital relationship (*P* = 0.007), dyspareunia (0.003) and mean age at which a sexual relationship was started (*P* = 0.006) (Table 1).

**Table 1:** Baseline characteristics of the participants

Variables	Case group (*n* = 136) Mean ± SD	Control group (*n* = 136) Mean ± SD	*P* value
Women age (year)	33.70 ± 8.44	35.70 ± 7.55	0.129**
Husband age (year)	38.38 ± 9.59	40.27 ± 7.45	0.061**
Age at first sexual intercourse (year)	21.77 ± 4.51	23.20 ± 4.08	0.006**
Duration of marriage (year)	9.60 ± 8.87	11.08 ± 8.44	0.150**
	Number (%)	Number (%)	
Women’s education			
Junior high school and below	13 (9.55)	6 (4.41)	< 0.001*
Senior high school	72 (52.94)	46 (33.82)	
College and above	51 (37.5)	84 (61.76)	
Spouses’education			
Junior high school and below	12 (8.82)	12 (8.82)	0.255*
Senior high school	72 (52.94)	59 (43.38)	
College and above	52 (38.23)	65 (47.79)	
Marital status			
Permanent marriage	129 (94.9)	136 (100)	
Temporary marriage	7 (5.1)	0	
Extramarital relationship			
Yes	8 (5.8)	0	0.007*
No	128 (94.2)	136 (100)	
Number of partner			
1	128 (94.2)	136 (100)	
2	8 (5.8)	0	
3	0	0	
≥ 4	0	0	
Sexual orientation			
Homosexual	0	0	
Heterosexual	136 (100)	136 (100)	
Bisexual	0	0	
Sexual style			
Vaginal	58 (42.8)	63 (46.3)	0.326*
Rectal	0	0	
Oral	0	0	
Vaginal and oral	8 (5.8)	15 (11.02)	
Vaginal and anal	48 (35.2)	39 (28.6)	
Vaginal, oral, and anal	22 (16.17)	19 (13.9)	
Condom use in intercourse			
Yes	46 (33.8)	56 (41.2)	0.260*
No	90 (66.2)	80 (58.8)	
Dyspareunia			
Yes	66 (48.5)	40 (29.4)	0.003*
No	75 (51.5)	96 (70.6)	
Start time of dyspareunia in case group			
Before genital warts	31 (47)	-	
After genital warts	35 (53)	-	

*Chi-square test was used; for variables with small cells (< 5), Fisher’s exact test was applied. In variables (marital status, number of partner, and sexual orientation) where, even after merging categories, there were very low frequencies, only descriptive statistics were reported. **Independent t-test

A comparison of the two groups revealed that there was a significant difference in the mean sexual distress score (*P* < 0.001) (Table 2). In the case group, 116 (85.29%) individuals and in the control group, 53 (38.97%) women had a score equal to or greater than 11 and experienced sexual distress disorder.

Table 2 shows that there was no statistically significant difference between the two groups in terms of the sexual avoidance score (*P* = 0.465). Since a higher score indicates more avoidance, the mean score of sexual avoidance was higher in the case group than in the control group.

**Table 2:** Comparison of the mean scores of sexual distress and sexual avoidance in women with and without genital warts

Group	Sexual distress score (Mean ± SD)	*P* value*	Sexual avoidance score (Mean ± SD)	*P* value*
With genital warts	26.16 ± 13.97	< 0.001	9.79 ± 4.0	0.465
Without genital warts	8.50 ± 6.42		9.48 ± 2.85	

* Independent t-test

The results of the generalized linear model showed that women with genital warts had a significantly higher sexual distress score of 17.28 units compared to individuals without genital warts (*P* < 0.001). Individuals with junior high school and below reported a significantly lower sexual distress score of 5.57 units compared to those with college and above education (0.036). Women with dyspareunia had a significantly higher sexual distress score of 4.10 units compared to those without the problem (*P* = 0.003). On average, with a one-year increase in women’s age at first sexual intercourse, the score of sexual distress increases by 0.453 units (*P* = 0.004). (Table 3).

**Table 3:** A generalized linear model for sexual distress

Variables	B	Std. Error	t	Sig.	95% Confidence Interval	
					Lower Bound	Upper Bound
Without genital warts	Ref					
With genital warts	17.279	1.358	12.724	< 0.001	14.605	19.953
College and above	Ref					
Senior high school	-0.322	1.380	-0.233	0.816	-3.040	2.396
Junior high school and below	-5.568	2.646	-2.104	0.036	-10.778	-0.359
Not having dyspareunia	Ref					
Having dyspareunia	4.100	1.355	3.026	0.003	1.433	6.767
Not having extramarital relationship	Ref					
Having extramarital relationship	2.267	3.871	0.586	0.559	-5.356	9.890
Age at the first sexual relationship	0.453	0.157	2.885	0.004	0.144	0.763

R Squared = 0.461, Adjusted R Squared = 0.443

Table 4 shows that none of the variables including having genital wart, education, dyspareunia, extra marital relationship and mean age at the first relationship showed a significant effect on sexual avoidance (*P* > 0.05).

**Table 4:** A generalized linear model for sexual avoidance

Parameter	B	Std. Error	t	Sig.	95% Confidence Interval	
					Lower Bound	Upper Bound
Without genital warts	Ref					
With genital warts	0.242	0.444	0.546	0.585	-0.631	1.116
College and above	Ref					
Senior high school	-0.243	0.451	-0.539	0.590	-1.131	0.645
Junior high school and below	1.136	0.864	1.314	0.190	-0.566	2.838
Not having dyspareunia	Ref					
Having dyspareunia	0.309	0.442	0.698	0.486	-0.562	1.180
Not having extramarital relationship	Ref					
Having extramarital relationship	-1.764	1.276	-1.383	0.168	-4.277	0.748
Age at the first sexual relationship	-0.049	0.052	-0.948	0.344	-0.151	0.053

R Squared = 0.052, Adjusted R Squared = 0.019

## Discussion

More than four-fifths of women with GWs experienced sexual distress in comparison to nearly two-fifths of women without GWs. The prevalence of sexual distress in different studies worldwide varies between 30% and 50% in women of reproductive age. The prevalence of sexual distress in Australian women aged 18–39 years was reported to be 50.6% [25]. This rate was measured at 31% in Slovenia [26]. A study in Iran reported that the prevalence of sexual distress was 27.2% in healthy women, 36.9% in women with sexual dysfunction, and 36.4% in people with hypoactive sexual desire disorder [23]. Another Iranian study revealed that 31.8% of women of reproductive age suffer from sexual distress [27]. Various conditions and diseases affect sexual function in women; among these diseases, STIs cause physical problems, body image changes, and psychological and interpersonal problems [28]. Body image is a factor associated with sexual desire disorders and can increase sexual distress by affecting sexual function [29]. A study on patients with genital psoriasis revealed that skin lesions led to a change in the patient’s positive body image and had a negative impact on sexual activity [17].

The results of a study in Iran revealed that healthy individuals reported higher levels of sexual satisfaction, self-confidence, self-esteem, and positive body image than did individuals experiencing sexual distress [30]. GWs, a disease characterized by skin lesions, can have a significant impact on a person’s body image and mental health. Studies have shown a correlation between body image and sexual distress, so individuals with GWs often report higher levels of sexual distress. On the other hand, having GWs can create challenges in a couple’s intimacy. It can weaken self-confidence, raise doubts about the sexual partner’s loyalty, and increase stress, anxiety, and dissatisfaction in the relationship. This can also increase sexual distress for the individual involved [9_11]. Findings of the current study showed that women experienced dyspareunia had a significantly higher sexual distress score. It was supported with Grano et al. study which indicated that the severity of dyspareunia significantly increases sexual distress, both through a direct effect and indirectly by reducing a person’s appreciation for their body’s functionality [31]. Furthermore, the current study found that women with lower educational attainment reported lower levels of sexual distress. This finding aligns with the research by Huberts et al., which also indicated that education level influences sexual distress, with more highly educated women experiencing greater sexual distress [32].

The prevalence of sexual avoidance, classified as a hypoactive sexual desire and arousal disorder, varies among different studies. A study conducted in Canada examined the prevalence and factors related to sexual avoidance disorder. The study revealed that the disorder affected 11.3% of women and 9.7% of men. Significant factors contributing to sexual avoidance include mental distress, dissatisfaction with sexual relationships, and individual stress and anxiety [33]. Another study conducted in Thailand focused on women with GWs reported that 72.4% of participants experienced hypoactive sexual desire disorder, 82.8% reported decreased sexual intercourse, 41.4% noted orgasmic disorder, and 57.1% had hypoactive sexual arousal disorder [10].

With respect to the factors influencing sexual avoidance, La Rocque et al. reported that individuals with a more negative body image tended to avoid sexual activity more frequently. Furthermore, they reported that sexual self-esteem, sexual satisfaction, and sexual desire play a mediating role in the relationship between body image and sexual avoidance [21]. GWs can affect their body image, leading to difficulties in sexual relationships, increasing stress and anxiety, suffering social stigma related to STIs, especially during the treatment process, impairing interpersonal relationships and marital satisfaction and leading to sexual avoidance [12, 33, 34]. In the present study, the mean score for sexual avoidance was greater in the case group; however, this difference was not statistically significant. It seems that the occurrence of sexual avoidance takes time, and first, unpleasant psychological consequences are created in people who are involved in GWs, and during a process, it leads to sexual avoidance. The greater degree of sexual distress in women with GWs could support the hypothesis in the current study. The mean onset time of GWs in women was nearly 5 months, and an assessment of sexual avoidance over a longer period after the appearance of GWs could provide more precise results regarding the evaluation of the psychosexual consequences.

Some potential limitations should be discussed. Nearly all the women in this study were married and heterosexual. Therefore, the results are not generalizable to single women with GWs with different sexual orientations. The use of convenience sampling limits generalizability. Women attending the clinic may differ from the general population in characteristics such as health status, level of sexual distress and avoidance, and health-related behaviors. Therefore, the results should be generalized with caution. This study did not consider women’s body image or sexual function in women or their partners. Future studies should consider these variables, which may affect sexual distress and sexual avoidance.

## Conclusion

The results revealed that women with GWs experienced more sexual distress. More than four-fifths of these women had sexual distress disorders. Sexual avoidance was not significantly different from that of women without GWs. These results could have important applications in the assessment of women with GWs. Healthcare providers should pay more attention to women’s sexual distress, imply appropriate interventions to diminish it and prevent the occurrence of undesirable psychosexual consequences such as sexual avoidance.

## Supplementary Information

Supplementary Material 1.

## Data Availability

The datasets used are available from the corresponding author upon reasonable request.

## Abbreviations

FSDS-R: Revised version of the female sexual distress scale
GWs: Genital warts
HPV: Human Papillomavirus
STI: Sexually transmitted infection

## References

[1] Ashique S, Hussain A, Fatima N, Altamimi MA. HPV pathogenesis, various types of vaccines, safety concern, prophylactic and therapeutic applications to control cervical cancer, and future perspective. Virusdisease. 2023;34(2):1–19. 37363362 10.1007/s13337-023-00824-z PMC10208188

[2] HPV and Cancer. 2022. https://www.cancer.gov/about-cancer/causes-prevention/risk/infectious-agents/hpv-and-cancer. Accessed 18 October 2023.

[3] Boda D, Docea AO, Calina D, Ilie MA, Caruntu C, Zurac S, et al. Human papilloma virus: apprehending the link with carcinogenesis and unveiling new research avenues (Review). Int J Oncol. 2018;52(3):637–55. 29393378 10.3892/ijo.2018.4256 PMC5807043

[4] Lisboa C, Santo I, Azevedo J, Azevedo L, Pista A, Dias C, et al. High prevalence of human papillomavirus on anal and oral samples from men and women with external anogenital warts: the HERCOLES study. Acta Derm Venereol. 2019;99(6):557–63. 30723872 10.2340/00015555-3136

[5] Khopkar US, Rajagopalan M, Chauhan AR, Kothari-Talwar S, Singhal PK, Yee K, et al. Prevalence and burden related to genital warts in India. Viral Immunol. 2018;31(5):346–51. 29717924 10.1089/vim.2017.0157

[6] Buenconsejo L, Kothari-Talwar S, Yee K, Kulkarni A, Lara N, Roset M, et al. Estimating the burden of illness related to genital warts in the philippines: A nationally representative cross-sectional study. Infect Agent Cancer. 2019;14(1):26. 31624494 10.1186/s13027-019-0240-y PMC6781391

[7] Jamdar F, Farzaneh F, Navidpour F, Younesi S, Balvayeh P, Hosseini M, et al. Prevalence of human papillomavirus infection among Iranian women using COBAS HPV DNA testing. Infect Agent Cancer. 2018;13:6. 29416557 10.1186/s13027-018-0178-5 PMC5784531

[8] Uzun SB, Sakin Ö, Çetin H, Şimşek EE. The effects of HPV test on Anxiety, emotion and depression in women. J Acad Res Med. 2020;10(2):149–54.

[9] Bennett KF, Waller J, McBride E, Forster AS, Di Gessa G, Kitchener H, et al. Psychosexual distress following routine primary human papillomavirus testing: a longitudinal evaluation within the english cervical screening programme. BJOG. 2021;128(4):745–54. 32783300 10.1111/1471-0528.16460 PMC8432156

[10] Parkpinyo N, Chayachinda C, Thamkhantho M. Factors associated with sexual dysfunction in women experiencing anogenital warts at Siriraj hospital. J Med Assoc Thai. 2020;103:359–64.

[11] El-esawy F, Ahmed H. Effect of genital warts on female sexual function and quality of life: an Egyptian study. Hum Androl. 2017;7:58–64.

[12] Nahidi M, Nahidi Y, Kardan G, Jarahi L, Aminzadeh B, Shojaei P, et al. Evaluation of sexual life and marital satisfaction in patients with anogenital wart. Actas Dermosifiliogr (Engl Ed). 2019;110:521–25. 30981378 10.1016/j.ad.2018.08.005

[13] Nick N, Torabizadeh C, Ghahartars M, Janghorban R. Adaptation of patients diagnosed with human papillomavirus: a grounded theory study. Reprod Health. 2021;18(1):236. 34702304 10.1186/s12978-021-01264-y PMC8547285

[14] Qaderi K, Mirmolaei ST, Geranmayeh M, Sheikh Hasani S, Farnam F. Iranian women’s psychological responses to positive HPV test result: a qualitative study. BMC Womens Health. 2021;21(1):128. 33771159 10.1186/s12905-021-01272-x PMC7995699

[15] Adeli M, Moghaddam-Banaem L, Shahali Sh, Ghandi N. Changes in sexual life in women with genital warts: A qualitative study. Health Educ Health Promot. 2022;10(3):593–602.

[16] American Psychiatric Association. Diagnostic and statistical manual of mental disorders: DSM-V. 5th ed. Arlington: American Psychiatric Publishing; 2013.

[17] Schielein MC, Tizek L, Schuster B, Ziehfreund S, Biedermann T, Zink A. Genital psoriasis and associated factors of sexual Avoidance - A People-centered Cross-sectional study in Germany. Acta Derm Venereol. 2020;100(10):adv00151. 32378723 10.2340/00015555-3509 PMC9137370

[18] Carvalheira A, Graham C, Stulhofer A, Traen B. Predictors and correlates of sexual avoidance among partnered older adults among Norway, Denmark, Belgium, and Portugal. Eur J Ageing. 2019;17(2):175–84. 32549872 10.1007/s10433-019-00540-y PMC7292841

[19] Popov SP, Mateva NG, Iliev YT, Dechev ID, Karalilova RV. Sexual fears and avoidant sexual behavior in medical students. Folia Med (Plovdiv). 2015;57(2):144–8. 26933786 10.1515/folmed-2015-0034

[20] McBride E, Marlow LAV, Forster AS, Ridout D, Kitchener H, Patnick J, et al. Anxiety and distress following receipt of results from routine HPV primary testing in cervical screening: the psychological impact of primary screening (PIPS) study. Int J Cancer. 2020;146(8):2113–21. 31251820 10.1002/ijc.32540 PMC7065242

[21] La Rocque CL, Cioe J. An evaluation of the relationship between body image and sexual avoidance. J Sex Res. 2011;48:397–408. 20672216 10.1080/00224499.2010.499522

[22] Derogatis L, Clayton A, Lewis-D’Agostino D, Wunderlich G, Fu Y. Validation of the female sexual distress scale-revised for assessing distress in women with hypoactive sexual desire disorder. J Sex Med. 2008;5(2):357–64. 18042215 10.1111/j.1743-6109.2007.00672.x

[23] Azimi Nekoo E, Burri A, Ashrafti F, Fridlund B, Koenig HG, Derogatis LR, Pakpour AH. Psychometric properties of the Iranian version of the female sexual distress scale-revised in women. J Sex Med. 2014;11(4):995–1004. 24641598 10.1111/jsm.12449

[24] Theiss JA, Estlein R. Antecedents and consequences of the perceived threat of sexual communication: A test of the relational turbulence model. Western J Communication. 2013;78(4):404–25.

[25] Zheng J, Skiba MA, Bell RJ, Islam RM, Davis SR. The prevalence of sexual dysfunctions and sexually related distress in young women: a cross-sectional survey. Fertil Steril. 2020;113(2):426–34. 32106994 10.1016/j.fertnstert.2019.09.027

[26] Starc A, Jukić T, Poljšak B, Dahmane R. Female sexual function and dysfunction: A Cross-National prevalence study in Slovenia. Acta Clin Croat. 2018;57:52–60. 30256011 10.20471/acc.2018.57.01.06 PMC6400359

[27] Hamzehgardeshi Z, Sabetghadam S, Pourasghar M, Khani S, Moosazadeh M, Malary M. Prevalence and predictors of sexual distress in married reproductive-age women: A cross-sectional study from Iran. Health Sci Rep. 2023;6(9):e1513. 37655267 10.1002/hsr2.1513 PMC10468024

[28] Kazemi Mojarad M, Khalatbari J, Moazedian A, Sotodehasl N. Investigating and determining psychological factors affecting women’s sexual function. J Adolesc Youth Psychol Stud. 2023;4(6):20–9.

[29] Ronghe V, Pannase K, Gomase KP, Mahakalkar MG. Understanding hypoactive sexual desire disorder (HSDD) in women: Etiology, Diagnosis, and treatment. Cureus. 2023;15(11):e49690. 38161863 10.7759/cureus.49690 PMC10757759

[30] Alizadeh A, Farnam F. Coping with dyspareunia, the importance of inter and intrapersonal context on women’s sexual distress: a population-based study. Reprod Health. 2021;18:161. 34321034 10.1186/s12978-021-01206-8 PMC8320204

[31] Grano C, Spinoni M, Porpora MG, Di Gesto C. Investigating the link between severity of dyspareunia and female sexual distress among a group of women with endometriosis: the mediating role of body functionality appreciation. J Sex Med. 2025;22(2):324–33. 39656634 10.1093/jsxmed/qdae175

[32] Huberts AS, Peeters NJMCV, Kaplan ZLR, van Linschoten RCA, Pastoor H, van der Woude CJ, et al. Dutch normative data of the sexual distress scale and the body image scale. Qual Life Res. 2023;32(10):2829–37. 37193810 10.1007/s11136-023-03434-w PMC10473982

[33] Lafortune D, Dussault É, Philibert M, Godbout N. Prevalence and correlates of sexual aversion: A Canadian Community-Based study. J Sex Med. 2022;19(8):1269–80. 35750625 10.1016/j.jsxm.2022.05.142

[34] Adeli M, Moghaddam-Banaem L, Shahali S. Sexual dysfunction in women with genital warts: a systematic review. BMC Womens Health. 2022;22(1):516. 36503516 10.1186/s12905-022-02073-6 PMC9743756

